# GDF-15 Predicts In-Hospital Mortality of Critically Ill Patients with Acute Kidney Injury Requiring Continuous Renal Replacement Therapy: A Multicenter Prospective Study

**DOI:** 10.3390/jcm10163660

**Published:** 2021-08-18

**Authors:** Jeong-Hoon Lim, Yena Jeon, Ji-Sun Ahn, Sejoong Kim, Dong Ki Kim, Jung Pyo Lee, Dong-Ryeol Ryu, Eun Young Seong, Shin Young Ahn, Seon Ha Baek, Hee-Yeon Jung, Ji-Young Choi, Sun-Hee Park, Chan-Duck Kim, Yong-Lim Kim, Jang-Hee Cho

**Affiliations:** 1 Department of Internal Medicine, Division of Nephrology, School of Medicine, Kyungpook National University, Kyungpook National University Hospital, 130 Dongdeok-ro, Jung-gu, Daegu 41944, Korea; ggumsuni@daum.net (J.-S.A.); hy-jung@knu.ac.kr (H.-Y.J.); jyss1002@hanmail.net (J.-Y.C.); sh-park@knu.ac.kr (S.-H.P.); drcdkim@knu.ac.kr (C.-D.K.); ylkim@knu.ac.kr (Y.-L.K.); 2 Department of Statistics, Kyungpook National University, Daegu 41566, Korea; yeahnah@naver.com; 3 Department of Internal Medicine, Seoul National University Bundang Hospital, Seongnam-si 13620, Korea; sejoong@snubh.org; 4 Department of Internal Medicine, Seoul National University College of Medicine, Seoul 08826, Korea; dkkim73@gmail.com (D.K.K.); nephrolee@gmail.com (J.P.L.); 5 Department of Internal Medicine, Seoul National University Boramae Medical Center, Seoul 07061, Korea; 6 Department of Internal Medicine, School of Medicine, Ewha Womans University, Seoul 07804, Korea; dr6302@gmail.com; 7 Division of Nephrology, Pusan National University School of Medicine, Busan 50612, Korea; sey-0220@hanmail.net; 8 Department of Internal Medicine, Korea University College of Medicine, Seoul 02841, Korea; ahnshinyoung712@gmail.com; 9 Department of Internal Medicine, Hallym University Dongtan Sacred Heart Hospital, Hwaseong 18450, Korea; haya2001@daum.net

**Keywords:** acute kidney injury, in-hospital mortality, continuous renal replacement therapy, growth differentiation factor-15

## Abstract

Growth differentiation factor-15 (GDF-15) is a stress-responsive cytokine. This study evaluated the association between GDF-15 and in-hospital mortality among patients with severe acute kidney injury (AKI) requiring continuous renal replacement therapy (CRRT). Among the multicenter prospective CRRT cohort between 2017 and 2019, 66 patients whose blood sample was available were analyzed. Patients were divided into three groups according to the GDF-15 concentrations. The median GDF-15 level was 7865.5 pg/mL (496.9 pg/mL in the healthy control patients). Baseline characteristics were not different among tertile groups except the severity scores and serum lactate level, which were higher in the third tertile. After adjusting for confounding factors, the patients with higher GDF-15 had significantly increased risk of mortality (second tertile: adjusted hazards ratio [aHR], 3.67; 95% confidence interval [CI], 1.05–12.76; *p* = 0.041; third tertile: aHR, 6.81; 95% CI, 1.98–23.44; *p* = 0.002). Furthermore, GDF-15 predicted in-hospital mortality (area under the curve, 0.710; 95% CI, 0.585–0.815) better than APACHE II and SOFA scores. Serum GDF-15 concentration was elevated in AKI patients requiring CRRT, higher in more severe patients. GDF-15 is a better independent predictor for in-hospital mortality of critically ill AKI patients than the traditional risk scoring system such as APACHE II and SOFA scores.

## 1. Introduction

Acute kidney injury (AKI) is a serious complication in hospitalized patients because it increases the mortality rates and the length of hospital stay [1,2]. In particular, AKI is more prevalent in critically ill patients in the intensive care unit (ICU) [3,4,5]. Continuous renal replacement therapy (CRRT) is the primary treatment for critically ill patients with severe AKI to correct biochemical imbalances and fluid status [6,7]. Despite the advances on CRRT that optimizes initiation timing and intensity, the mortality rate of patients in the ICU with severe AKI has not improved yet [8,9]. Early diagnostic indicators that accurately reflect the patient status and predict prognosis are needed to plan a treatment regimen fit for each patient and to improve outcomes in critically ill patients requiring CRRT.

Several scoring systems can predict the mortality rate of critically ill patients in ICU, such as Assessment and Chronic Health Evaluation II (APACHE II) and the Sequential Organ Failure Assessment (SOFA) scoring systems; in addition, biomarkers such as neutrophil gelatinase-associated lipocalin (NGAL), kidney injury molecule-1 (KIM-1), and c-terminal fibroblast growth factor 23 (cFGF-23) can also predict the outcome of patients in ICU with AKI [10,11,12]. However, the predictive power of the indicators for mortality is not sufficient, particularly among severe AKI patients requiring CRRT [13,14,15], because the etiology of AKI varies and clinical courses are usually not expected due to the effects of high rates of morbidity and mortality [16].

Growth differentiation factor-15 (GDF-15) is a highly divergent member of the transforming growth factor (TGF)-β superfamily. It is also known as macrophage inhibiting cytokine 1 (MIC-1) [17], placental transformation growth factor (PTGF-β), ref. [18] prostate derived factor (PDF) [19], placental bone morphogenetic protein (PLAB) [20], or nonsteroidal anti-inflammatory drug-activated gene-1 (NAG-1) [21]. GDF-15 is a stress-responsive cytokine and increases in various pathologic conditions such as heart failure, chronic kidney disease, diabetes, chronic obstructive pulmonary disease, and solid cancers [22,23,24,25,26,27,28,29]. GDF-15 is recently identified as a prognostic biomarker for cardiovascular diseases [22,30,31,32], end-stage kidney disease [25,33], acute respiratory distress syndrome [34], and sepsis [35]. Therefore, the present study aims to evaluate the role of GDF-15 as a predictor of in-hospital mortality in critically ill patients with severe AKI requiring CRRT.

## 2. Materials and Methods

### Study Population

This multicenter prospective study analyzed the data of patients enrolled in a randomized controlled trial, named VolumE maNagement Under body composition monitoring in critically ill patientS on CRRT (VENUS) trial. The VENUS trial involved critically ill patients with AKI aged over 18 years who require CRRT; patients with chronic kidney disease or imminent death were excluded. The detailed criteria for enrollment and study design of VENUS trial are shown in our previous study [36]. Among the enrolled 126 patients in VENUS trial between July 2017 and September 2019, 59 were excluded from the present study due to withdrawal of consent (*n* = 2), insufficient or no blood samples at the time of CRRT initiation (*n* = 50), and early death after CRRT initiation (*n* = 8) (Supplementary Figure S1). Finally, a total of 66 patients who were survived more than 48 h after initiation of CRRT were included in this study. For negative controls, nine blood samples from healthy donors were collected to compare GDF-15 levels. This study was conducted under the ethical principles of the Declaration of Helsinki and approved by the Institutional Review Board of Kyungpook National University Hospital (KNUH 2018-08-018), Seoul National University Bundang Hospital, Seoul National University Hospital, Seoul National University Boramae Medical Center, and Ewha Womans University Mokdong Hospital. Written informed consent for study participation was obtained from all the patients before inclusion. This study was registered with the ClinicalTrials.gov (NCT03330626) and the Clinical Research Information Service (CRiS) at the Korea Centers for Disease Control and Prevention (KCT0002534). This study was conducted in accordance with the guidelines of the 2013 Declaration of Helsinki.

## 3. Data Collection

Baseline clinical information of study participants including age, sex, body mass index (BMI), Charlson Comorbidity Index (CCI), cause of AKI, comorbidities, blood pressure, SOFA score, APACHE II score, and use of intensive care were collected at the time of CRRT initiation. Septic AKI was defined when the patient met systemic inflammatory response syndrome criteria and suspected infection [37]; ischemic AKI was defined when the patient had hemodynamic instability without sepsis. Laboratory data including white blood cell count, hemoglobin, blood urea nitrogen, creatinine, sodium, potassium, lactate, albumin, and high-sensitivity C-reactive protein (hs-CRP) were also collected on the same day. Estimated glomerular filtration rate (eGFR) was calculated using the Modification of Diet in Renal Disease equation [38]. The prescription of CRRT was determined and followed by the responsible nephrologists, and target clearance was assessed at the initiation of CRRT. The outcome of the study was in-hospital mortality, and survival status was investigated at 28, 60, and 90 days.

## 4. Measurement of Growth Differentiation Factor-15 (GDF-15)

The blood samples were collected before CRRT on the day of initiation using heparin or EDTA as an anticoagulant. After centrifugation for 15 min at 1000 × g within 30 min after collection, plasma samples were assayed immediately, and aliquots stored at ≤−20°C. The level of GDF-15 in the samples was measured using a commercial kit (human GDF-15 Quantikine Immunoassay, R&D Systems, Minneapolis, MN, USA) according to the manufacturer’s instructions. Briefly, this is the quantitative sandwich enzyme immunoassay technique. A human GDF-15 specific monoclonal antibody has been pre-coated onto a microplate. Standards and samples are pipetted into the wells; all GDF-15 present is bound by the immobilized antibody. After washing to remove any unbound substrates, an enzyme-linked polyclonal antibody specific for human GDF-15 is added to the wells. Then, rewashing to remove any unbound antibody-enzyme reagent and a substrate solution is added to the wells. Finally, color develops proportionally to the amount of initial GDF-15 bound. After color development stopped, the intensity of the color is measured.

## 5. Statistical Analysis

Patients were stratified into tertile according to the distribution of GDF-15 levels. The Kolmogorov–Smirnov test was used to analyze the normal distribution of variables. Continuous variables are expressed as mean ± standard deviation or median (interquartile range) based on distribution, and categorical variables are expressed as numbers (percentage). The one-way analysis of variance with post hoc Scheffe test or Kruskal–Wallis test with Dunn’s post hoc test was used to compare continuous variables, and Pearson chi-square tests or Fisher’s exact tests were used to compare categorical variables, as appropriate. The Kaplan–Meier analysis with log-rank test and Cox proportional hazard analysis were performed to compare in-hospital mortality. Clinically significant variables were selected for the multivariable Cox regression model, such as age, sex, SOFA score, and serum lactate level. In addition, for the analysis of GDF-15 as a quantitative variable, the relationship between GDF-15 and in-hospital mortality was evaluated using a Cox proportional hazard model with restricted cubic spline functions to capture potential non-linear effects. Univariable and multivariable linear regressions were used to evaluate associated factors with GDF-15; significant variables in univariable analysis and clinically important variables were selected for multivariable analysis, such as age, sex, CCI, SOFA score, and serum lactate level. The receiver operating characteristic (ROC) analysis was performed to evaluate each parameter’s ability to predict mortality; the ability was assessed by area under the curve (AUC). AUC values were compared using a method proposed by DeLong et al. [39] and MedCalc Statistical Software version 19.6.1 (MedCalc Software Ltd., Ostend, Belgium). The net reclassification improvement (NRI) and integrated discrimination improvement (IDI) were calculated to evaluate the added predictive ability on mortality of the GDF-15 beyond that of the conventional severity scores such as APACHE II and/or SOFA. Statistical analyses were performed using SPSS Statistics for Windows version 22.0 (IBM Corp., Armonk, NY, USA). A *p* value less than 0.05 was considered statistically significant.

## 6. Results

### 6.1. Baseline Characteristics at the Time of Continuous Renal Replacement Therapy (CRRT) Initiation

The baseline characteristics of the entire study participants and each tertile group by GDF-15 level are shown in Table 1. The mean age was 67.7 ± 14.3 years and 47 (71.2%) were male. Age, sex, BMI, and mean arterial pressure did not differ among tertile groups. The cause of AKI was sepsis in half (*n* = 33, 50.0%) and 27 patients (40.9%) were ischemic AKI. SOFA score was higher in the third tertile than the first tertile, and APACHE II score was also higher in the third tertile than the second tertile (both *p* < 0.05). Intensive care rates such as mechanical ventilation and vasopressor use at the time of CRRT were similar among tertile groups. Median value of serum GDF-15 was 7856.5 pg/mL (interquartile range [IQR], 5759.4–9435.6) and each tertile group was as follows: tertile 1: 5187.3 pg/mL (IQR, 4678.0–5798.5); tertile 2: 7856.5 pg/mL (IQR, 6904.0–8525.0); tertile 3: 10,064.1 pg/mL (IQR, 9371.8–10,497.2). Most of the laboratory findings did not differ except serum lactate. Serum lactate level was significantly higher in the third tertile than the first tertile or second tertile (both *p* < 0.05). Target clearance of CRRT was also not different among groups.

**Figure 1:**
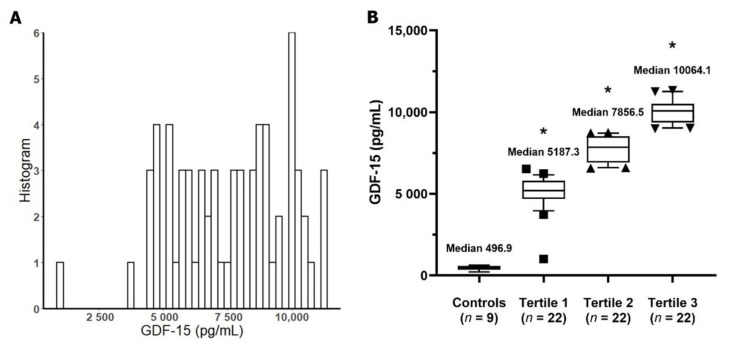
Growth differentiation factor-15 (GDF-15) concentrations in study patients. (**A**) Distribution of GDF-15. (**B**) GDF-15 concentration in the tertile healthy individual groups. * *p* < 0.001 vs. healthy control group. Abbreviation: GDF-15, growth differentiation factor-15.

Figure 1 displays GDF-15 distribution among study patients. We measured the GDF-15 concentration of healthy control group. The mean age of the healthy control was 38.7 ± 5.2 years, and 55.6% were male. Most of the measured GDF-15 concentrations in AKI patients were evenly distributed between 4000 and 11,000 pg/mL. GDF-15 concentrations in healthy control subjects were significantly lower compared to each tertile group (all *p* < 0.001).

### 6.2. Association between GDF-15 and In-Hospital Mortality

**Figure 2:**
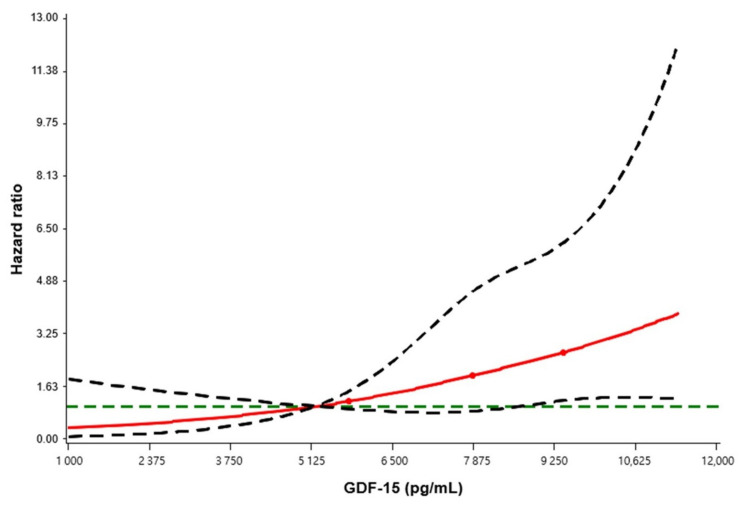
Association between GDF-15 concentration and in-hospital mortality hazard ratio by restricted cubic spline regression model. The reference value is the median of the first tertile (5187.3 pg/mL). The red line indicates the estimated hazard ratio; the dashed green line indicates the reference line of null hypothesis that the hazard ratio is 1; the dashed black lines indicate the lower and upper 95% confidence limits. Abbreviation: GDF-15, growth differentiation factor-15.

**Figure 3:**
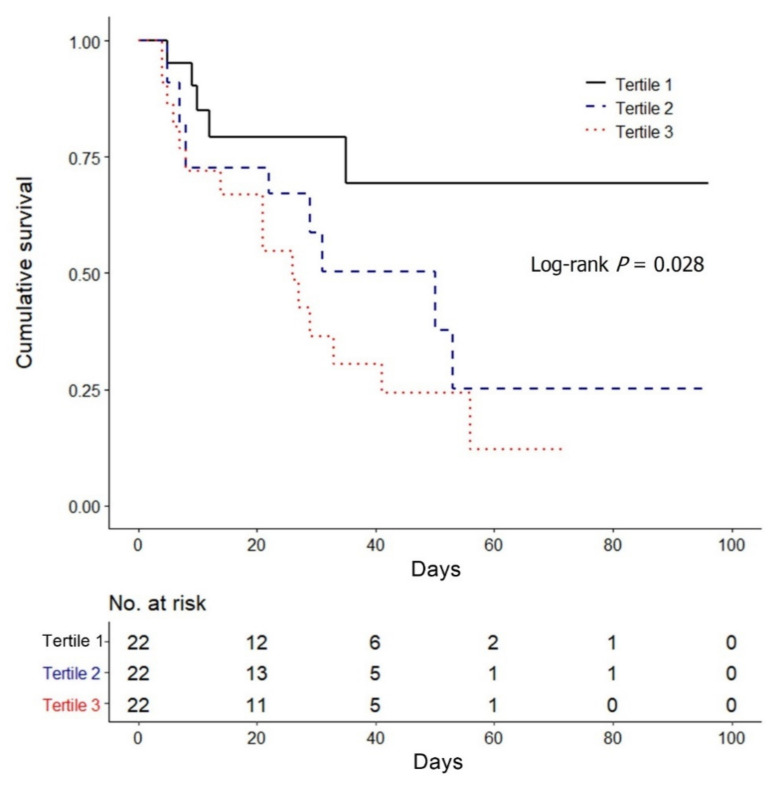
Kaplan–Meier survival curve for in-hospital mortality by GDF-15 tertiles. Abbreviation: GDF-15, growth differentiation factor-15.

A Cox regression analysis using restricted cubic splines was used to evaluate the relationship between GDF-15 and in-hospital mortality (Figure 2). The reference value was the median value of the first tertile (5187.3 pg/mL), and the result revealed that higher GDF-15 levels is associated with an increased in-hospital mortality in AKI patients treated CRRT. The Kaplan–Meier survival analysis also revealed the consistent result that the in-hospital mortality was significantly higher in the tertile groups with high GDF-15 levels than the first tertile (log-rank *p* = 0.028) (Figure 3).

In the univariable Cox regression analysis, the third tertile group of GDF-15 revealed a significantly increased risk of in-hospital mortality compared to the first tertile (model 1: hazard ratio [HR], 3.65; 95% confidence interval [CI], 1.33–10.08; *p* = 0.012) (Table 2). The mortality rate in the higher GDF-15 tertile groups were significantly higher after adjusting for age, sex, and CCI (model 2: second tertile: adjusted HR [aHR], 3.26; 95% CI, 1.04–10.22; *p* = 0.042; third tertile: aHR, 4.70; 95% CI, 1.59–13.90; *p* = 0.005), and for age, sex, CCI, SOFA score, and serum lactate (model 3: second tertile: aHR, 3.67; 95% CI, 1.05–12.76; *p* = 0.041; third tertile: aHR, 6.81; 95% CI, 1.98–23.44; *p* = 0.002) (Table 2).

### 6.3. Comparison of GDF-15 for Predicting In-Hospital Mortality with Other Prognostic Markers

**Figure 4:**
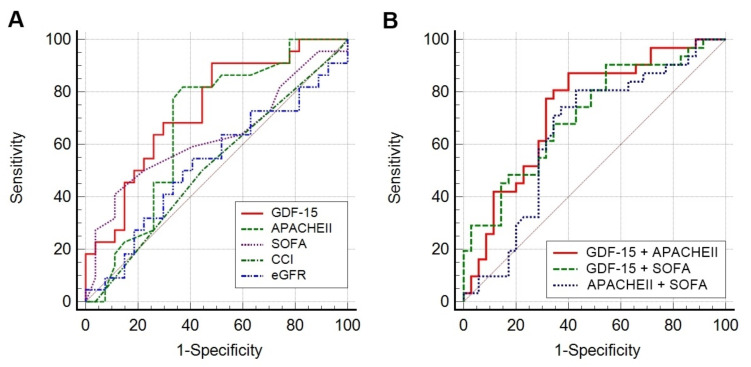
Receiver operating characteristic curves of prognostic predictors for in-hospital mortality. (**A**) Results for single variables. (**B**) Results for combined variables. The AUC values are as follows: GDF-15 (0.710); APACHE II (0.624); SOFA (0.584); CCI (0.519); eGFR (0.527); GDF-15 + APACHE II (0.735); GDF-15 + SOFA (0.712); APACHE II + SOFA (0.645). Abbreviations: GDF-15, growth differentiation factor-15; APACHE, acute physiology and chronic health evaluation; SOFA, sequential organ failure assessment; CCI, Charlson Comorbidity Index; eGFR, estimated glomerular filtration rate.

Figure 4 shows the ROC curves of prognostic parameters for in-hospital mortality. Among the single parameters, GDF-15 showed the highest AUC value (0.710; 95% CI, 0.585–0.815) followed by APACHE II score (0.624; 95% CI, 0.497–0.741), SOFA score (0.584; 95% CI, 0.456–0.704), CCI (0.519; 95% CI, 0.393–0.644), and eGFR at the time of CRRT (0.527; 95% CI, 0.379–0.671); among the combined parameters, integrated GDF-15 and APACHE II score showed the highest AUC value (0.735; 95% CI, 0.612–0.836) followed by integrated GDF-15 and SOFA score (0.712; 95% CI, 0.588–0.817), and integrated APACHE II score and SOFA score (0.645; 95% CI, 0.518–0.759).

The AUC values of GDF-15 alone, integrated GDF-15 and APACHE II score, integrated GDF-15 and SOFA score, and integrated GDF-15, APACHE II score, and SOFA score were significantly larger than 0.5 (all *p* < 0.05) (Table 3). The NRI for the addition of GDF-15 to conventional severity scores significantly improved predictability when added to the APACHE II score; in addition, the NRI for the combination of GDF-15 and SOFA score showed an increase of predictability in trend. IDI also revealed that the addition of GDF-15 significantly improves the predictive power of the individual model such as APACHE II score, SOFA score, and APCHE II + SOFA score.

### 6.4. Associated Factors of GDF-15

In the univariable linear regression analysis, SOFA score and serum lactate level had a positive correlation with GDF-15 level (both *p* < 0.001) and APACHE II score showed a trend of a positive correlation with GDF-15 (*p* = 0.059, Table 4). Both SOFA score and serum lactate level were also independent predictors for GDF-15 after adjusting for clinically important parameters including age, sex, and CCI in the multivariable linear regression analysis (both *p* < 0.01).

## 7. Discussion

This prospective multicenter study demonstrated that serum GDF-15 concentration is a strong predictor for in-hospital mortality among critically ill patients with AKI who require CRRT. All the AKI patients showed higher serum GDF-15 concentration than healthy control and GDF-15 level was closely associated with traditional severity indices in critically ill patients. These findings support that GDF-15 concentrations could reflect severity in critically ill AKI patients and effectively predict mortality in these patients.

AKI increases mortality in hospitalized patients and the close relationship is strongly dependent on the severity of AKI [40,41]. Among AKI patients treated with CRRT, the mortality has been reported up to 40–50% in previous studies [42,43]. The high mortality rate is associated with CRRT being applied to patients with multiple severe comorbidities such as sepsis, shock, and other organ failure [44,45]. Various laboratory biomarkers and several scoring systems using clinical and laboratory parameters have been developed to estimate risk of mortality in patients with severe AKI. However, they were difficult to apply or the results were not satisfactory [10,16,46]. In this respect, serum GDF-15, which is easy to measure, could be a useful biomarker to predict mortality in critically ill AKI patients.

GDF-15 plays a role as a stress-induced cytokine with diverse actions at various organs, vascular system, and neutrophils. It also regulates cellular autonomy linked to cellular senescence and apoptosis [47,48,49,50]. Inflammation, oxidative stress, and injury induce GDF-15 rise [51], stimulation of interleukin (IL)-1β, tumor necrosis factor (TNF)-α, macrophage colony-stimulating factor, angiotensin II, and TGF-β upregulates GDF-15 expression [47]. In healthy individuals, serum GDF-15 concentration ranges from 200 to 1150 pg/mL and increases with age [52,53,54]. The receptors and downstream mediators of GDF-15 have not yet been identified in most tissues; thus, the role and pathophysiologic mechanism of GDF-15 remains elusive in most diseases [46].

Many recent clinical studies have confirmed the predictive value of GDF-15 for mortality and clinical courses in various diseases, such as cardiovascular disease, heart failure, and kidney diseases [25,30,32,54,55]. We demonstrated that the GDF-15 concentration is also an effective predictor for mortality in critically ill patients with AKI; causes of AKI were various, including sepsis and ischemia. A previous study has reported similar results that GDF-15 concentration is a predictor for mortality in critically ill sepsis patients [35]. Compared to the study, all patients in this study had severe kidney failure and much higher GDF-15 concentrations than the previous study. It is consistent in that GDF-15 levels are closely associated with organ dysfunctions [22,25,30,31,35,49,56,57].

There are several studies that evaluate the protective effect and potential utility of GDF-15 in AKI. Zimmers et al. showed GDF-15 was an early mediator of the injury response in AKI animal model [58]. The murine septic AKI model revealed that GDF-15 deficiency augments inflammatory response and exacerbates kidney injury, while over-expression of GDF-15 protects the kidney [59]. A recent experimental study also demonstrated the renoprotective and immunomodulatory effects of GDF-15 in the AKI mouse model invoked by ischemic reperfusion injury [60]. Similarly, other studies have reported that GDF-15 dampens inflammatory reactions [35,61]. These results consistently support the anti-inflammatory and renoprotective effects of GDF-15 in AKI. Furthermore, Guenancia et al. showed the potential of GDF-15 as a biomarker for diagnosing AKI [62]; the pre-operative serum GDF-15 was associated with post-operative AKI in patients with cardiac bypass surgery. 

Several human and animal studies reported the association of GDF-15 with chronic kidney disease. Higher serum GDF-15 was associated with diabetic kidney disease and occurrence of incident chronic kidney disease [63,64]. GDF-15 also showed renoprotective effects in type 1 and 2 diabetes mouse model by decreasing of interstitial inflammation and fibrosis [65]. This suggests that GDF-15 is involved in the prognosis and pathogenesis of various kidney diseases as well as AKI.

Serum GDF-15 concentration was strongly correlated with lactate level and SOFA score. GDF-15 level in severe sepsis was significantly associated with organ dysfunction markers such as creatinine (kidney) and bilirubin (liver), and disease severity markers such as lactate, hs-CRP, APACHE II score and SOFA score [25,33,35,57]. In the present study, critically ill AKI patients showed much higher GDF-15 levels compared to the healthy controls. The concentration of GDF-15 was associated with SOFA score (reflects degree of organ dysfunction) and lactate level (reflects disease severity with macro/microcirculation) [35,66]. This suggests that GDF-15 predicts mortality by reflecting the degree of organ dysfunction and disease severity in critically ill AKI patients.

Serum GDF-15 concentration at the time of CRRT showed better performance in predicting in-hospital mortality among critically ill AKI patients compared to conventional scoring systems. Both APACHE II and SOFA scores are representative predictors for mortality in ICU patients. Many studies have demonstrated the accurate predictive power of these scores for mortality in critically ill patients [67,68,69,70]. However, severe AKI commonly causes electrolyte imbalances and fluid imbalances, making these scores difficult in predicting mortality in critically ill AKI patients [13,14,15]. Our study demonstrated that GDF-15 had a practical predictive value for mortality even as a single factor. Furthermore, the combination of GDF-15 with conventional severity scores, such as APACHE II score or SOFA score provides additional predictability. Even when added to the combination model of APACHE II and SOFA scores, predictive power was improved. Therefore, serum GDF-15, combined with the traditional risk scoring systems, can be used as a useful biomarker to increase predictive performance.

Though CRRT has not been shown to improve mortality and renal recovery compared to intermittent hemodialysis in critically ill patients with AKI, CRRT is considered the standard of care at many centers for AKI associated with hemodynamic instability [71,72]. The importance of this study is that it demonstrated the excellent prediction power of GDF-15 for in-hospital mortality in critically ill AKI patients and identified the potential of GDF-15 as a biomarker predicting mortality. However, there are several limitations to this study. First, we only measured GDF-15 at the time of CRRT initiation and did not measure GDF-15 concentration before AKI. Because GDF-15 is elevated in both critically ill and AKI status, it is difficult to assess the dominant factor for GDF-15 increase and the causal relationship with GDF-15 elevation in this study. Second, the serial changes of serum GDF-15 concentration were not measured during hospitalization, so that we cannot assess the relationship between clinical course and the change of GDF-15 levels. Third, we compared the GDF-15 concentration with the healthy control group, of which the mean age was much younger than that of AKI groups. As serum GDF-15 concentration increases with age in healthy individuals, the comparison between healthy and AKI groups has limitations. However, the young healthy control group did not affect the main results in that the present study aimed to evaluate the predictive value of GDF-15 for mortality among critically ill AKI patients. Fourth, although the GDF-15 concentration may reflect the overall severity of diseases, we did not compare GDF-15 levels to non-AKI patients with similar overall severity (e.g., matched by SOFA or APACHE II scores). Therefore, it is difficult to know whether the predictive value of GDF-15 is specific to AKI or not. Finally, the patients who died within 48 h after CRRT initiation were excluded from the study. The clinical information for the patients could not be obtained according to the study design of the VENUS trial. This may limit the generalizability and further studies are required for the impact of GDF-15 on the early critical period.

In conclusion, serum GDF-15 concentration was elevated in critically ill patients with AKI who require CRRT. GDF-15 concentration had a strong correlation with the lactate level and SOFA score. GDF-15 is a better independent predictor for in-hospital mortality of critically ill AKI patients than the traditional risk scoring system such as APACHE II and SOFA scores.

## Figures and Tables

**Table 1:** Baseline characteristics at the time of continuous renal replacement therapy (CRRT) initiation.

Variables	All (*n* = 66)	Tertile 1 (*n* = 22)	Tertile 2 (*n* = 22)	Tertile 3 (*n* = 22)	*p*
Age, y	67.7 ± 14.3	68.7 ± 15.0	68.3 ±11.6	66.1 ± 16.5	0.819
Sex, male, *n* (%)	47 (71.2)	16 (72.7)	17 (77.3)	14 (63.6)	0.596
BMI, kg/m^2^	25.0 ± 3.5	25.2 ± 3.2	24.5 ± 2.3	25.3 ± 4.6	0.732
MAP, mmHg	79.0 ± 14.4	80.4 ± 14.8	78.6 ± 16.8	77.9 ± 11.8	0.839
Cause of AKI					0.313
Septic	33 (50.0)	8 (36.4)	11 (50.0)	14 (63.6)	
Ischemic	27 (40.9)	10 (45.5)	10 (45.5)	7 (31.8)	
Others	6 (9.1)	4 (18.2)	1 (4.5)	1 (4.5)	
Comorbidities, *n* (%)					
Hypertension	26 (39.4)	9 (40.9)	9 (40.9)	8 (36.4)	0.939
Diabetes	27 (40.9)	9 (40.9)	13 (59.1)	5 (22.7)	0.058
CHF	9 (13.6)	3 (13.6)	2 (9.1)	4 (18.2)	0.901
CVA	6 (9.1)	4 (18.2)	1 (4.5)	1 (4.5)	0.348
Malignancy	23 (34.8)	5 (22.7)	7 (31.8)	11 (50.0)	0.154
CCI	2.6 ± 1.5	3.1 ± 1.5	2.3 ± 1.1	2.5 ± 1.7	0.233
SOFA score	11.0 ± 3.8	9.2 ± 2.9 ^a^	11.7 ± 4.0 ^a,b^	12.0 ± 4.0 ^b^	0.026
APACHE II score	35.4 ± 8.6	34.3 ± 8.0 ^a,b^	33.7 ± 7.7 ^a^	39.0 ± 8.2 ^b^	0.044
Ventilator apply, *n* (%)	51 (77.3)	14 (63.6)	17 (77.3)	20 (90.9)	0.097
Vasopressor use, *n* (%)	54 (81.8)	16 (72.7)	19 (86.4)	19 (86.4)	0.559
Admission to CRRT, d	1.0 (1.0, 3.3)	1.0 (0, 3.0)	1.0 (0, 4.0)	1.0 (0, 3.0)	0.857
Laboratory findings					
GDF-15, pg/mL	7856.5 (5759.4, 9435.6)	5187.3 (4678.0, 5798.5)	7856.5 (6904.0, 8525.0)	10,064.1 (9371.8, 10,497.2)	<0.001
WBC count, ×10^3^/μL	14.7 ± 9.2	13.1 ± 8.2	16.4 ± 10.1	14.7 ± 9.7	0.511
Hb, g/dL	10.1 ± 2.4	9.8 ± 2.6	10.8 ± 2.2	9.5 ± 2.2	0.137
BUN, mg/dL	48.5 (33.3, 67.8)	60.5 (35.3, 80.3)	48.5 (39.0, 63.5)	35.0 (27.5, 63.0)	0.179
Creatinine, mg/dL	3.0 (2.1, 4.2)	3.0 (2.2, 5.4)	3.3 (2.2, 4.0)	2.2 (1.6, 3.5)	0.088
eGFR, mL/min/1.73 m^2^	17.9 (12.6, 29.1)	17.3 (10.3, 29.2)	18.2 (12.1, 26.6)	25.2 (16.2, 40.3)	0.131
Sodium, mEq/L	139.0 (136.0, 142.0)	141.0 (136.8, 143.0)	136.5 (131.8, 141.3)	139.0 (137.0, 141.0)	0.070
Potassium, mEq/L	4.2 (3.8, 5.4)	4.2 (3.6, 5.4)	4.3 (3.9, 4.9)	4.9 (3.6, 5.5)	0.637
Lactate, mEq/L	4.8 (2.4, 8.5)	2.8 (1.4, 6.0) ^a^	4.0 (2.5, 5.5) ^a^	10.4 (5.6, 13.8) ^b^	<0.001
Albumin, g/dL	2.6 (2.4, 3.1)	2.6 (2.3, 2.9)	2.7 (2.5, 3.1)	2.6 (2.3, 3.1)	0.489
hs-CRP, mg/dL	10.3 (4.6, 18.1)	12.8 (6.0, 17.8)	5.4 (4.5, 15.5)	10.3 (2.0, 18.7)	0.622
Target clearance, mL/min	32.9 ± 6.3	32.8 ± 5.5	32.6 ± 5.8	33.4 ± 7.6	0.393

The different superscripts (a, b) denote significant differences between groups not sharing the same superscript at the 0.05 level. Abbreviations: CRRT, continuous renal replacement therapy; BMI, body mass index; MAP, mean arterial pressure; AKI, acute kidney injury; CHF, congestive heart failure; CVA, cerebrovascular accident; CCI, Charlson Comorbidity Index; SOFA, sequential organ failure assessment; APACHE, acute physiology and chronic health evaluation; GDF-15, growth differentiation factor-15; WBC, white blood cell; Hb, hemoglobin; BUN, blood urea nitrogen; eGFR, estimated glomerular filtration rate; hs-CRP, high sensitivity C-reactive protein.

**Table 2:** Association between GDF-15 and mortality in Cox proportional hazards analysis.

Variables	Model 1 ^†^		Model 2 ^‡^		Model 3 ^§^	
	HR (95% CI)	*p*	aHR (95% CI)	*p*	aHR (95% CI)	*p*
GDF-15						
Tertile 1	Reference		Reference		Reference	
Tertile 2	2.39 (0.83–6.91)	0.107	3.26 (1.04–10.22)	0.042	3.67 (1.05–12.76)	0.041
Tertile 3	3.65 (1.33–10.08)	0.012	4.70 (1.59–13.90)	0.005	6.81 (1.98–23.44)	0.002

^†^ Model 1: unadjusted relative risk; ^‡^ model 2: adjusted for age, sex, and CCI; ^§^ model 3: adjusted for model 2 plus SOFA score and serum lactate level. Abbreviations: GDF-15, growth differentiation factor-15; HR, hazard ratio; CI, confidence interval; aHR, adjusted hazard ratio; CCI, Charlson Comorbidity Index; SOFA, sequential organ failure assessment.

**Table 3:** Comparison of AUC and predictive power of prognosis predictors for in-hospital mortality.

Variables	AUC (95% CI)	*p* ^a^	NRI	*p*	IDI	*p*
GDF-15	0.710 (0.585–0.815)	0.001				
APACHE II	0.624 (0.497–0.741)	0.080	Reference		Reference	
APACHE II + GDF-15	0.735 (0.612–0.836)	<0.001	0.490	0.040	0.103	0.007
SOFA	0.584 (0.456–0.704)	0.249	Reference		Reference	
SOFA + GDF-15	0.712 (0.588–0.817)	<0.001	0.418	0.083	0.114	0.005
APACHE II + SOFA	0.645 (0.518–0.759)	0.039	Reference		Reference	
APACHE II + SOFA + GDF-15	0.727 (0.601–0.854)	0.003	0.361	0.136	0.103	0.007
CCI	0.519 (0.393–0.644)	0.783				
eGFR at the time of CRRT	0.527 (0.379–0.671)	0.754				

^a^ The pairwise comparison of AUC values between the parameter and 0.5. Abbreviations: AUC, area under the curve; CI, confidence interval; NRI, net reclassification improvement; IDI, integrated discrimination improvement; GDF-15, growth differentiation factor-15; APACHE, acute physiology and chronic health evaluation; SOFA, sequential organ failure assessment; CCI, Charlson Comorbidity Index; eGFR, estimated glomerular filtration rate; CRRT, continuous renal replacement therapy.

**Table 4:** Associated factors for GDF-15 in linear regression analysis.

Variables	Univariate			Multivariate		
	B (SE)	*β*	*p*	B (SE)	*β*	*p*
Age	−6.3 (19.4)	−0.04	0.748	28.0 (25.3)	0.18	0.273
Sex (ref: F)	−453.6 (607.3)	−0.09	0.458	53.9 (542.3)	0.01	0.921
BMI	−11.2 (80.2)	−0.02	0.889			
CCI	−240.0 (187.5)	−0.16	0.205	−375.2 (246.8)	−0.25	0.134
Hypertension	−188.8 (564.7)	−0.04	0.739			
Diabetes	−528.2 (557.8)	−0.12	0.347			
SOFA	247.3 (66.5)	0.42	<0.001	216.2 (65.0)	0.37	0.002
APACHE II	60.4 (31.4)	0.23	0.059			
Lactate	142.3 (36.3)	0.45	<0.001	121.4 (34.5)	0.38	0.001
Albumin	−22.7 (54.2)	−0.05	0.676			
eGFR	18.8 (17.2)	0.14	0.280			
hs-CRP	−7.9 (32.1)	−0.03	0.807			

Abbreviations: GDF-15, growth differentiation factor-15; B, unstandardized regression coefficient; SE, standard error; β, standardized coefficient; BMI, body mass index; CCI, Charlson Comorbidity Index; SOFA, sequential organ failure assessment, APACHE, acute physiology and chronic health evaluation; eGFR, estimated glomerular filtration rate; hs-CRP, high sensitivity C-reactive protein.

## Data Availability

The datasets generated during and/or analysed during the current study are available from the corresponding author on reasonable request.

## Supplementary Materials

The following are available online at https://www.mdpi.com/article/10.3390/jcm10163660/s1, Figure S1: Flow diagram. Abbreviations: CRRT, continuous renalre.

## Trial Registration

ClinicalTrials.gov, NCT03330626. Registered 6 November 2017; Clinical Research Information Service (CRiS) at the Korea Centers for Disease Control and Prevention, KCT0002534. Registered 16 November 2017.
