# Supplementary figures and images for: GDF-15 Predicts In-Hospital Mortality of Critically Ill Patients with Acute Kidney Injury Requiring Continuous Renal Replacement Therapy: A Multicenter Prospective Study
Source: J Clin Med. 2021 Aug 18;10(16):3660. doi: 10.3390/jcm10163660 (PMC8397174; doi:10.3390/jcm10163660)

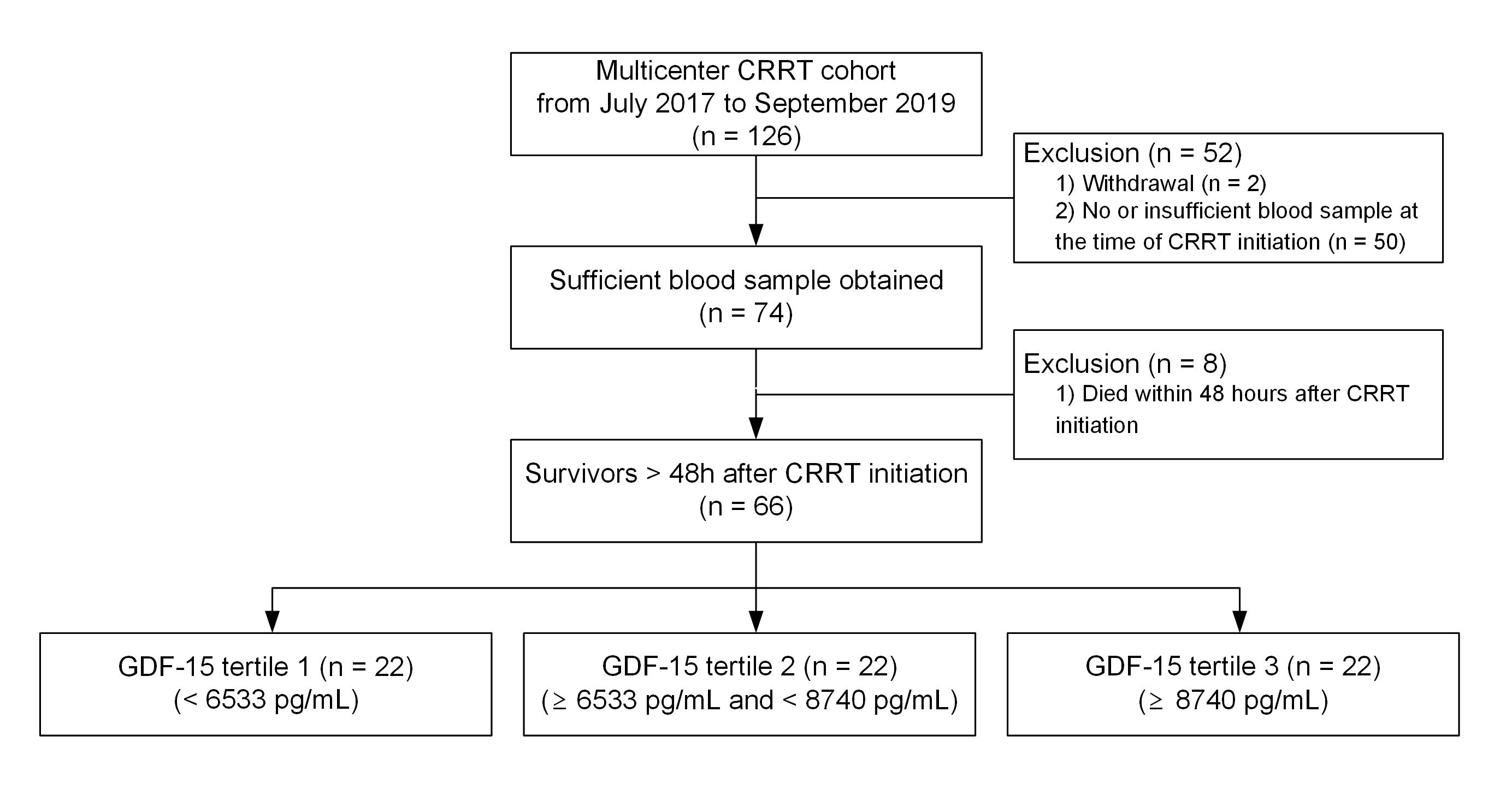

Supplement: Supplementary file 1 [file jcm-10-03660-s001.zip › Figure S1_revised.jpg]
